# Supplementary material for: Nestin Modulates Airway Smooth Muscle Cell Migration by Affecting Spatial Rearrangement of Vimentin Network and Focal Adhesion Assembly
Source: Cells. 2022 Sep 29;11(19):3047. doi: 10.3390/cells11193047 (PMC9562664; doi:10.3390/cells11193047)
Supplement: Supplementary file 1 [file cells-11-03047-s001.zip › cells-1943315-supplementary.pdf]

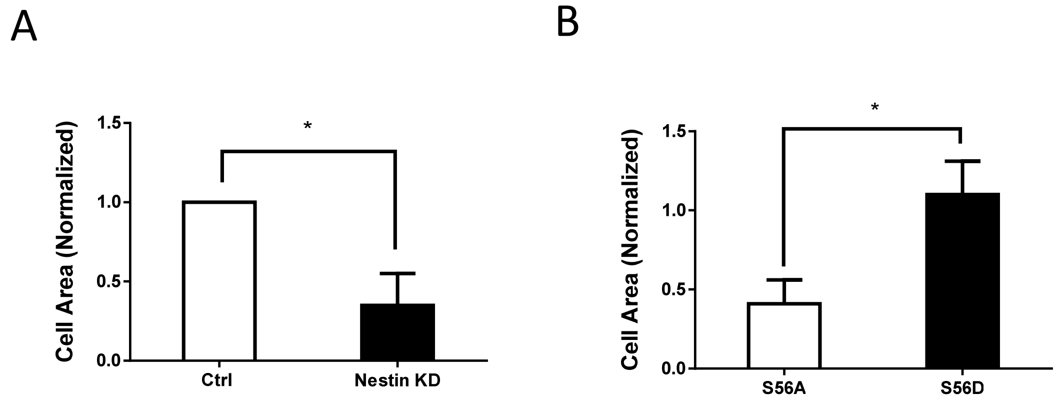

Figure S1. Role of nestin and its phosphorylation in cell spreading. A. Nestin KD reduces the areas of whole cell body. **B.** Expression of S56D vimentin increases cell body area of nestin KD cells. Cell body areas are normalized to control cells. Data are mean ± SE (n = 25-28 cells). * $P < 0.05$

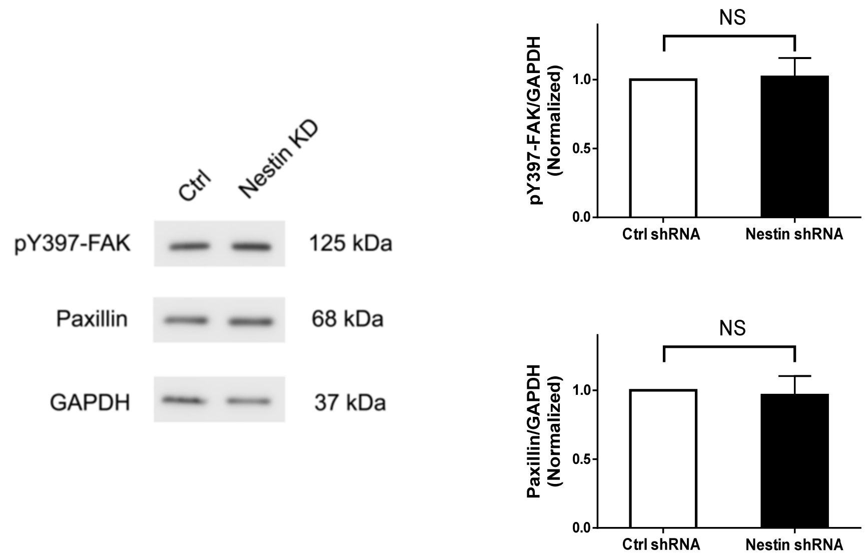

Figure S2. Nestin KD does not affect expression of pY397-FAK and paxillin in cells. Protein expression of human airway smooth muscle (HASM) cells stably expressing control (Ctrl) shRNA or nestin shRNA was evaluated by immunoblotting. Data are mean values of experiments from 4 batches of cell culture. Error bars indicate SE. NS, not significant.

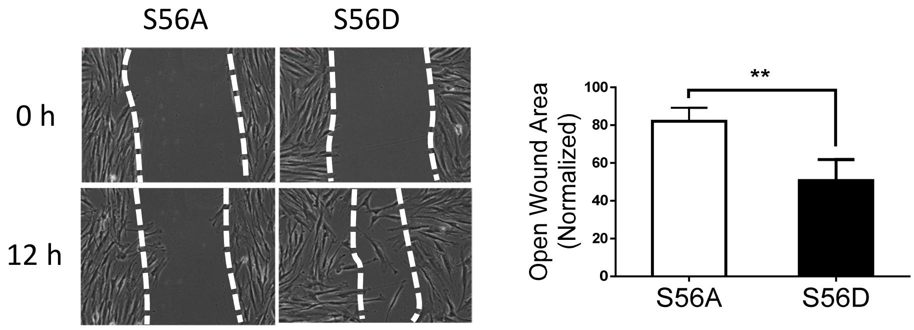

Figure S3. S56D enhances the migration of nestin KD cells. Nestin KD cells were transfected with S56A or S56D vimentin. Cell migration was evaluated using the wound healing assay. Data are mean ± SE (n = 5). P < 0.01.
